# Core and Shell Contributions to the Phonon Spectra of CdTe/CdS Quantum Dots

**DOI:** 10.3390/nano13050921

**Published:** 2023-03-01

**Authors:** Volodymyr Dzhagan, Nazar Mazur, Olga Kapush, Oleksandr Selyshchev, Anatolii Karnaukhov, Oleg A. Yeshchenko, Mykola I. Danylenko, Volodymyr Yukhymchuk, Dietrich R. T. Zahn

**Affiliations:** 1 V. Lashkaryov Institute of Semiconductors Physics, National Academy of Sciences of Ukraine, 03028 Kyiv, Ukraine; 2 Physics Department, Taras Shevchenko National University of Kyiv, 01601 Kyiv, Ukraine; 3 Semiconductor Physics, Chemnitz University of Technology, D-09107 Chemnitz, Germany; 4 Center for Materials, Architectures, and Integration of Nanomembranes (MAIN), Chemnitz University of Technology, D-09107 Chemnitz, Germany; 5 Frantsevich Institute for Problems of Materials Science, National Academy of Sciences of Ukraine, 03142 Kyiv, Ukraine

**Keywords:** CdTe nanocrystals, core/shell nanocrystals, infrared absorption, Raman spectra, phonons

## Abstract

The parameters of the shell and interface in semiconductor core/shell nanocrystals (NCs) are determinant for their optical properties and charge transfer but are challenging to be studied. Raman spectroscopy was shown earlier to be a suitable informative probe of the core/shell structure. Here, we report the results of a spectroscopic study of CdTe NCs synthesized by a facile route in water, using thioglycolic acid (TGA) as a stabilizer. Both core-level X-ray photoelectron (XPS) and vibrational (Raman and infrared) spectra show that using thiol during the synthesis results in the formation of a CdS shell around the CdTe core NCs. Even though the spectral positions of the optical absorption and photoluminescence bands of such NCs are determined by the CdTe core, the far-infrared absorption and resonant Raman scattering spectra are dominated by the vibrations related with the shell. The physical mechanism of the observed effect is discussed and opposed to the results reported before for thiol-free CdTe Ns as well as CdSe/CdS and CdSe/ZnS core/shell NC systems, where the core phonons were clearly detected under similar experimental conditions.

## 1. Introduction

Semiconductor nanocrystals (NCs) or quantum dots (QDs) have been intensively investigated for more than three decades due to the unique interplay of quantum confinement effects and surface physics and chemistry. Properties of such NCs can be easily tuned by synthesis, leading to numerous promising properties [1,2,3]. In any of the perspective applications, the properties of the NC surface or the interface in case of core/shell NCs are determinant, primarily for their photoluminescence (PL) [3,4,5], as well as for charge and energy transfer phenomena [4,5,6,7,8]. In particular, an interplay of the shell thickness, effect of alloying (either targeted by the synthesis or occurring in an uncontrollable manner) at the core/shell interface, and the magnitude of residual strain in both core and shell is determinant for the NC properties, but hard to be measured [4,5,9,10]. Resonant Raman scattering by phonons has proved to be an efficient tool for solving this problem [9,10,11,12,13,14,15,16]. It benefits from well-separated vibrational frequencies of typical pairs of core and shell materials (e.g., CdSe and CdS), from the possibility of selective excitation of core or shell phonons by tuning the excitation wavelength, as well as from the known phonon behavior with variation of lattice strain or alloy composition [17,18,19,20]. The most often studied core/shell NC system is CdSe/CdS [6,13,14,16,21,22], few works on CdSe/ZnS were reported [11,23], while no Raman study of CdTe/CdS NCs were reported so far. The authors of Ref. [24] reported the spectra of NCs assuming initially a CdTe/CdS core/shell structure, but after they had been subject to sintering at temperatures from 200 to 500 °C they presumably lost both their nanocrystalline form and core/shell structure. The spectra of bare CdTe NCs were studied in some works [25], although much less intensively compared to CdSe and CdS ones (see recent reviews [11,12]). In view of the continuous interest of researchers in the employment of the optical properties of CdTe NCs in general [1,2] and those synthesized in aqueous solution using thiol stabilizer in particular [1,3,26,27,28,29,30], deeper insight into their structural and vibrational properties is required.

Here, we report the results of a Raman study of CdTe NCs synthesized by a facile scalable route in water, using thioglycolic acid (TGA) as a stabilizer of the NCs in the colloid [26,27,28]. We show that using thiol during the synthesis results in the formation of a CdS shell around the CdTe core NCs. Even though the UV-vis absorption and PL spectra of such NCs are determined by the CdTe core, the far-infrared absorption and resonant Raman spectra are dominated by the vibrations related with the shell. The possible physical mechanism of the observed effects is suggested and compared with the results reported before for bare CdTe as well as CdSe/CdS and CdSe/ZnS core/shell NC systems, where the core phonons were clearly detected under similar experimental conditions.

## 2. Materials and Methods

The synthesis and optical properties of aqueous colloidal CdTe NCs of such type as those used in this work were partially described by us earlier [26,27,28]. Briefly, the process of CdTe synthesis was accomplished at 20 °C in argon atmosphere with the use of the following reagents: 0.1 M solution of CdI_2_ (reagent grade), thioglycolic acid (TGA) (99%), and electrochemically prepared hydrogen telluride. Deionized water was used as the dispersion medium. CdI_2_ was dissolved in water, and TGA was added under stirring, followed by adjusting the pH to 11 by dropwise addition of NaOH solution. The duration of the synthesis was 1 min. As the exhaust of the reactor may contain unreacted rests of H_2_Te, they were neutralized by passing the exhaust through 0.1 ± 0.05 mol/L aqueous solution of NaOH under 40 W white light illumination. The vibrational spectroscopic results in this paper are representative; qualitatively similar Raman and IR features were observed for NC samples synthesized at different conditions within this facile and scalable synthesis approach, at least for those where the intensity of the PL background allowed registration of Raman spectra of acceptable quality. The difference between the spectra was a minor variation in the relative intensities of the Raman bands, which do not influence the general and qualitative effects discussed in this paper.

The Raman spectra were obtained using a 1 mW 457 nm single-longitudinal-mode solid-state laser for excitation, a single-stage spectrometer (MDR-23, LOMO, St. Petersburg, Russia) with a spectral resolution of 3 cm^−1^ for dispersion, and TE-cooled CCD detector (Andor iDus 420, Oxford Instruments, Abingdon, UK). Raman spectra were acquired from the dried NC films deposited by drop-casting of as-synthesized NC solutions without purification onto a Si substrate. Optical absorption and PL spectra were obtained using a Silver Nova 25 BWI6 spectrometer (StellarNet, Tampa, FL, USA) and a RF-1501 spectrofluorometer (Shimadzu, Kyoto, Japan), correspondingly. Transmission electron microscopy (TEM) images were obtained with a JEM-2100F (JEOL, Tokyo, Japan) microscope; an acetone suspension of the NCs underwent an ultrasonic treatment and was then deposited onto a copper grid covered with an ultrathin carbon film. Dynamic light scattering (DLS) measurements were performed with a particle sizer NanoBrook Omni (Brookhaven Instruments, Holtsville, NY, USA) equipped with a 532 nm laser. Infrared spectra were obtained using a Bruker 80v FTIR spectrometer (Bruker, Billerica, MA, USA) in reflectance mode. XPS measurements were performed at a spectral resolution of 0.5 eV using an ESCALAB 250Xi X-Ray Photoelectron Spectrometer (Thermo Scientific, Waltham, MA, USA) equipped with a monochromatic Al K_α_ (hν = 1486.7 eV) X-ray source. To prevent charging, the samples were measured using a built-in charge compensation system. Spectra deconvolution and quantification were performed using the Avantage Data System (Thermo Scientific, Waltham, MA, USA). The energy scale was calibrated with an accuracy of ±0.05 eV using the binding energies (BE) of Au4f_7/2_ at 83.95 eV, Ag3d_5/2_ at 368.20 eV, Cu2p_3/2_ at 932.60 eV, and the Fermi edge at 0.00 eV, measured on in situ cleaned metal surfaces, as well as using adventitious carbon C1s BE of 284.8 eV as the common internal standard [31,32,33].

## 3. Results

The methods of colloidal synthesis of CdTe NCs using thioglycolic acid (TGA) as a stabilizer of the NCs in the colloid is a facile and scalable route that is broadly used to obtain highly fluorescent NCs at a relatively low temperature in water [26,27,28,29,30,34]. By varying the duration of the thermal treatment during the synthesis, the electrical current, the pH of the medium or the TGA concentration and some other parameters, the spectral position of the PL can be tuned and its intensity maximized. The characterization of the optical properties of the CdTe NCs obtained by this method was partially reported by us earlier [26,27,28]. Here, we report the representative vibrational properties of such NCs, studied by resonant Raman spectroscopy and FTIR spectroscopy in the far-infrared range.

**Figure 1:**
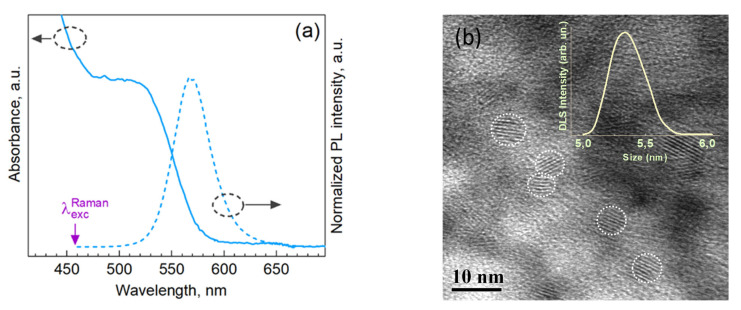
Optical absorption (solid line) and PL (dashed line) spectra (**a**) and a representative TEM image (**b**) of the CdTe-TGA NCs. The inset shows the DLS data on the NC size measured in the solution.

In UV-vis spectra, the CdTe-TGA NCs reveal a characteristic absorption feature due to the lowest-energy interband transition and near-bandgap (excitonic) PL (Figure 1a), which are strongly shifted toward shorter wavelengths with respect to the fundamental absorption edge of bulk CdTe (820 nm) [35,36]. The mean NC size can be evaluated from the spectral position of the step-like feature in UV-vis spectrum, which is related with the lowest-energy transition between the discrete/quantized electronic states in the NCs. Based on the dependence of this absorption peak position on the CdTe NC size well-studied in the literature [37], the average diameters of the NCs studied in this work are in the range of 2.5–3.5 nm.

A representative TEM image of a sample corroborates this assessment and proves the formation of highly crystalline NCs in the above size range, although more clearly visible as individual particles in the images are the largest NCs from the ensemble, 4–5 nm, while the smaller NCs mostly occur as aggregates formed during drying of the solvent on the TEM grid (Figure 1b). The results of the dynamic light scattering (DLS) measurements (inset in Figure 1b) reveal a mean NC size about 5.3 nm, which is in a reasonable agreement with both the optical and TEM results, because DLS provides the hydrodynamic size of the NCs, i.e., the diameter of the inorganic core plus the thickness of the layer of stabilizing (TGA) molecules. Both TEM and DLS data prove that the UV-vis spectrum in Figure 1a is determined by the quantum-confined CdTe.

**Figure 2:**
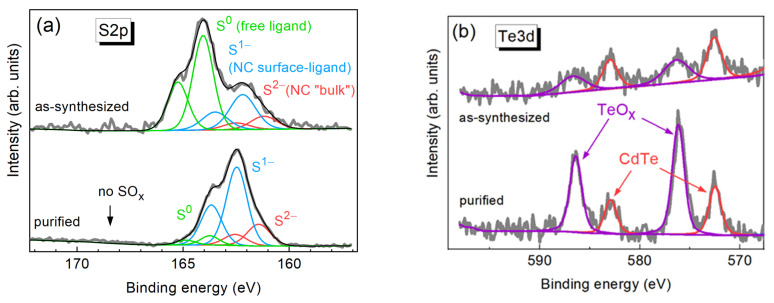
High-resolution XPS spectra of CdTe-TGA NCs in the range of S2p (**a**) and Te3d (**b**) core levels for the as-synthesized NCs (upper spectra) and after purification from excess ligands (lower spectra).

A more detailed insight in the structure of the CdTe-TGA NCs was obtained from the XPS measurements. In particular, it is known from previous studies of thiol-stabilized NCs that the S2p range is informative on the chemical states and bonding of the thiol ligand [31]. A pair of representative S2p spectra of the NCs is shown in Figure 2a for as-synthesized NCs and for NCs after purification of the colloidal solution from excess ligands used during the synthesis. A satisfactory fit of the spectra can be obtained with three components (i.e., three S2p_3/2_/S2p_1/2_ doublets). The assignment of these components can be made based on our previous works on other sulfide NCs stabilized with TGA [31] and XPS studies of other authors on thiol-stabilized CdTe NCs [38]. The low binding energy (BE) component peaked at 161.2/162.4 eV for as-synthesized sample and at 161.5/162.8 eV for NCs subjected to purification can be attributed to fully coordinated sulfide ions S^2−^. Observing this component indicates the formation of a CdS shell around the CdTe core, which was also found in some previous studies on thiol-stabilized CdTe [38] and CdSe NCs [21]. Noteworthy is that we did not detect any formation of a sulfide shell for Cu-Zn-Sn-Te (CZTTe) NCs synthesized under similar mild conditions and using the same stabilizer, TGA [32]. Therefore, one can conclude that the sort of cations in the NC core is an important factor that affects the formation of the CdS shell and or its alloying with the core.

The second doublet, at 162.2/163.4 eV in the as-synthesized and at 162.4/163.6 eV in the purified sample, can be attributed to the surface-bound TGA molecule, i.e., sulfur atoms that are actually a part of the NC surface [21,31,39]. The third component, at 164.0/165.2 eV in the as-synthesized and at 163.8/165.0 eV in the purified sample, is due to the free ligand, i.e., excess TGA molecules used during the synthesis but not bound to the surface in the resultant NC solution. This binding energy is typical for elemental sulfur or S-S, S-C, and S-H groups in organic compounds [33]. In the work on Cu-Zn-Sn-S (CZTS) NCs [31] it was assumed that a S-S bridged disulfide may form from TGA during the reduction of Cu^2+^ to Cu^+^ (2Cu^2+^ + 2RSH = 2Cu^+^ + RSSR + 2H^+^) [40]. In our case of CdTe NCs, the formation of such a bond is less probable, however cannot be completely ruled out due to possible oxidation of TGA by air oxygen. Nevertheless, the significant reduction of the intensity of the third component upon purification while preserving the first two of them (Figure 2a), observed earlier also for other types of NCs [31], additionally supports the above assignment of all three S2p components.

It should be noted that we do not register an additional feature at 168–170 eV, which was observed in some works, where thiol was used as a ligand [21], and attributed to a SO_x_ species on the NC surface. Therefore, no noticeable oxidation of the sulfur atoms on NC surface occurs under the given synthesis conditions and film preparation conditions. Note that no SO_x_ species were detected for CZTS and CZTTe NCs synthesized in a similar way using the same ligand, TGA [31,32].

The XPS spectra in the Te3d binding energy (BE) range shown in Figure 2b contain several components (i.e., Te3d_5/2_/Te3d_3/2_ doublets). The first one, with a BE around 572.5/582.9 eV, is mostly related to Te^2−^ in the NC lattice (Te3d_5/2_ at 572.5–572.7 eV for CdTe [33,41]). It should be noted that the chemical shift of Te^0^ (Te3d_5/2_ 572.85–572.94 eV [41]) is below the experimental resolution of 0.5 eV, so a certain contribution of elemental tellurium cannot be completely excluded and does not contradict the Raman scattering data discussed below. The second component at BE 576.0/586.4 eV can be assigned to tellurium oxides (TeO_2_, Te3d_5/2_ 576.1 eV [41]). The oxide-related component gets stronger upon removal of the stabilizer excess, which can be understood based on the expected partial loss of the bound and free ligands removed by the purification. The latter can sacrificially react with air oxygen and thus serve as an antioxidant reagent. The detection of oxidized Te atoms indicates that the CdS shell is not continuous on a small fraction of the NCs, allowing a partial oxidation of the surface Te atoms. Alternatively, one may assume that the observed TeO_x_ signal is caused not by the Te belonging to the CdTe NCs but by some minor content of elemental Te that may remain/form in the solution during the synthesis. However, we would expect this contribution to be reduced after purification, while we observe an opposite effect, which is more reasonable for the oxidized Te to be related with the CdTe NC surface. Therefore, based on the results obtained so far and the high intensity of the excitonic PL and absence of the defect/surface related PL, we suggest that most of the NCs have a CdTe/CdS core/shell structure, while a small fraction of CdTe NCs does not have a complete or thick enough shell to withstand surface Te oxidation.

**Figure 3:**
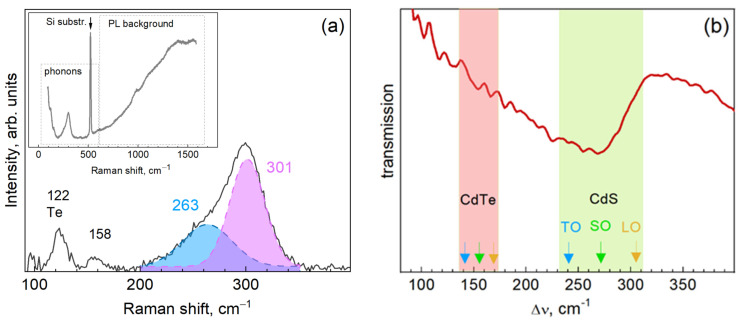
Representative Raman (**a**) and infrared (**b**) spectra of the CdTe-TGA NCs. For the Raman spectrum, the PL background was subtracted and the main feature fitted with two components. In the inset to (**a**), the as-measured (raw) spectrum is shown with the ranges indicated where NC phonons and PL background are expected. In (**b**), the position of the bulk TO and LO frequencies are indicated by arrows, and the range of possible SO positions by a shaded areas for CdS and CdTe.

Further understanding of the structure of the CdTe-TGA NCs was obtained from studying their vibrational (phonon) spectra by means of infrared absorption and resonant Raman spectroscopy. Representative Raman spectra of the CdTe NCs are shown in Figure 3a along with a fit of the strongest feature with two components after background subtraction. The as-measured (raw) spectrum is shown in the inset, where the ranges of the NC phonons and PL background are indicated.

Based on the previous studies of bulk CdTe [42,43] and CdTe NCs [19,44,45,46,47,48,49], the main features expected in the resonant Raman spectra of our NCs are the LO phonon peak around 170 cm^−1^ and its overtones. However, as we can see from the spectra in Figure 3a instead of the expected peak we observe the main peak around 300 cm^−1^ and weaker features at 122 and 158 cm^−1^. The peak at 122 cm^−1^ can be attributed to elemental Te, which is known for its large Raman cross-section that enables measurable Raman signals even at tiny concentrations in some matrix [50] or monolayer coverage on a surface [42,43,51]. This observation is in line with the XPS results discussed above.

The main feature, spread between 200 and 350 cm^−1^ (Figure 3a), can be attributed to the vibration of cadmium sulfide, because CdS has the TO mode at 240 cm^−1^ and the LO mode at 305 cm^−1^ [52,53]. Therefore, the formation of a CdS shell on the CdTe NCs can be concluded from the Raman spectra and is corroborated by the XPS data discussed above. The lineshape of the CdS band in the Raman spectra of the present CdTe/CdS NCs is very similar to that of the CdSe/CdS NCs studied earlier [13,14,21,54]. Deconvolution of the latter in the previous studies was dependent on the thickness of the CdS layer and degree of interdiffusion with the core. For a thin shell, not exceeding 1–2 nm, it was fitted as a convolution of a CdS-like SO mode (at 262–267 cm^−1^) and a CdS-like mode of the alloyed layer (CdS_x_Se_1-x_) at the interface (at 285–288 cm^−1^) [13,14]. For a thick shell, 3 nm or more, in addition to these two components, a LO mode of the shell material was clearly identified (at 295–298 cm^−1^) [13,14]. Therefore, in the spectrum of CdTe/CdS NCs in Figure 3a the band at 301 cm^−1^ can be related with the LO phonon of the shell, while the low-frequency shoulder can be a superposition of the CdS SO mode and the CdS-like vibration of the alloyed CdS_x_Te_1-x_ interface layer.

Phonon Raman spectra allow us to exclude that the detected CdS phase is formed as separate NCs. It was shown in numerous previous studies of II-VI NCs [11,25] that the Raman spectra of “free-standing” II-VI NCs, even as small as <2 nm, reveal the overtone of the (first-order) LO phonon mode (2LO). In view of the rather strong intensity of the CdS-related first order feature in the spectra of the present NCs (Figure 3a), if the CdS phase were formed as separate NCs, this should result in a measurable 2LO around 600 cm^−1^. However, this is not the case in our spectra. Furthermore, the separately formed CdS NCs would exhibit their own PL, most likely the surface-related broad band PL centered at 500–600 nm. Such PL is typical for TGA-stabilized CdS NCs with ligands synthesized in mild aqueous conditions [39] but not observed in our case, where only the sharp and symmetric band of the CdTe core related excitonic (or near-bandgap) emission is observed (Figure 1a). In addition, the PL spectra not showing any intensity in the red or near-infrared range, which could be related with traps on the CdTe NC surface, is a further indication that the surface of these (CdTe) NCs is well passivated by an inorganic (CdS) shell.

The 2.7 eV (457.8 nm) laser line used for excitation of the Raman spectra is resonant with the electronic transition of the CdTe/CdS NC as a whole, because they exhibit the absorption features due to the lowest interband transition at 520–530 nm and the higher-energy transitions form a continuous absorption at shorter wavelengths (Figure 1a). In Ref. [55], it was shown that in CdSe/CdS core/shell NCs, the CdSe phonons are relatively strong when exciting in resonance with the lower excitonic transitions, for which the hole is largely localized in the CdSe core. However, the CdSe core phonon becomes nearly undetectable when the energy of the excitation quantum is more than 0.6 eV above the lowest exciton energy of the NCs, where both electrons and holes are largely localized in the CdS shell. The CdS phonon Raman cross section exhibits a maximum 0.6–0.7 eV above the lowest exciton and then decreases at higher energies [55]. These results are corroborated by previous works of our and other groups reporting for the CdSe/CdS and CdSe/ZnS core/shell NCs that by changing the λ_exc_ from 514.5 to 457.8 nm the Raman bands related with Cd-S vibrations in the CdS shell and the alloyed interface of the CdSe/CdS or CdSe/ZnS NCs were enhanced [11,14]. Noteworthy is that the ZnS shell phonon has never been observed [23], while the observed band at 270–280 cm^−1^ was attributed either to a Cd-S alloy mode of the intermixed interface [14] or to the ZnS TO mode [23]. One can assume that the thin shell of this wide-bandgap material is far from resonance even with the shorter excitation wavelength commonly available for the Raman experiments, 325 nm or ≈3.8 eV, because the ZnS SO mode was also not observed [23]. Noteworthy is that the LO phonon peaks of ZnS (as well as ZnO) shell were detected at λ_exc_ = 325 nm for CuInS_2_/ZnS and CuInS_2_/ZnO, respectively [56], most likely due to larger shell thicknesses, but the peculiarities of the localization of the electronic wavefunctions and electron-phonon coupling (EPC) may also play a role.

It should be noted that even under conditions of strong alloying with the shell material (CdS), the LO phonons of the core (CdTe) should be observable, because they were shown to give comparable contribution with the CdSe counterpart both in other core/shell systems [57] and in homogeneously alloyed NCs [19,20,58]. Not observing the LO peak of the CdTe core in CdTe/CdS NCs may, therefore, be related with the peculiarities of the electron-phonon coupling in the system. The intensity of resonant Raman scattering by LO phonons in CdTe and CdS is determined by the Fröhlich mechanism of the electron-phonon coupling. Therefore, the dominance of the CdS peak in the spectrum of the present CdTe/CdS NCs can be due to the much higher constant of EPC of CdS, 0.73, than for CdTe, 0.29, resulting in a relatively small Raman cross-section in the latter [18,42,51]. Note that a Raman spectrum dominated by the shell phonons and no core phonons being observed was reported earlier only in giant shell (≈7–13 nm) CdSe/CdS NCs [22]. Moreover, it was shown in Ref. [59] that the EPC strength is considerably higher for an alloy with intermediate compositions compared with those of the pure components. This is an additional argument for a stronger Raman signal of an alloyed shell compared to that of the core.

The weak feature at 158 cm^−1^ in the spectra of our CdTe-TGA NCs is too far from the CdTe LO (170 cm^−1^) and TO (≈140 cm^−1^ [60]). Based on its intermediate position it could be assigned to the SO mode, as it has been observed for bare CdTe NCs [25], as well as for other II-VI NCs between the corresponding TO and LO frequencies [25]. However, the SO mode has been commonly not observed alone, but as a weak shoulder of the LO mode [44,49]. Therefore, the 158 cm^−1^ feature in the spectra in the present work is more likely to be the CdTe-like mode of the alloyed interface. The CdS_x_Te_1-x_ ternary system is known to exhibit a “two-mode” behavior with CdS- and CdTe-like longitudinal optical phonon modes [52]. The CdS-like LO and TO phonons converge to the triply degenerate vibrational local mode of S in CdTe at 259 cm^−1^ [52]. The CdTe-like LO mode of the CdS_x_Te_1-x_ alloy exhibits a very weak dependence on *x*, and the local Te mode in bulk CdS is expected only 12 cm^−1^ lower, 160 cm^−1^, than the LO phonon frequency of bulk CdTe, 172 cm^−1^ [52]. Based on the results reported earlier for the CdSe/CdS system [14], the alloyed interface layer may be as thin as ≈1 nm. Therefore, a downward shift of the phonons in such a thin layer due to phonon confinement can reasonably explain the attribution of the 158 cm^−1^ feature to the CdTe-like mode of the alloyed CdS_x_Te_1-x_ interface of the CdTe/CdS NCs.

The spectra of IR absorption in the range of phonons (Figure 3b) corroborate the Raman results: the main absorption band is peaked at nearly the same position (260 cm^−1^) as the Raman component attributed to the CdS SO mode. The prevalence of the SO modes in the IR absorption of II-VI NCs is in agreement with our previous results obtained for CdSe/CdS NCs [14]. This property can be explained by the mixed TO/LO nature of the SO mode in the NCs, while the partially transversal nature of the vibration ensures their efficient IR absorption. The absorption shoulder extending toward smaller wavenumbers may be related with the contribution of the CdTe SO and TO modes, expected around 150–160 cm^−1^ and 140 cm^−1^, respectively [61].

## 4. Conclusions

CdTe NCs synthesized by a facile scalable route in water, using thioglycolic acid (TGA) as a stabilizer of the NCs in the colloid were investigated by X-ray photoelectron spectroscopy (XPS) as well as Raman and infrared vibrational spectroscopies. We detected that using thiol during the synthesis results in the formation of a CdS shell around the CdTe core NCs. Even though the spectral position of the optical absorption and PL bands of such NCs are determined by the CdTe core, the resonant Raman scattering and infrared absorption spectra are dominated by the vibrations related with the shell. Indications of alloying in the phonon spectra are similar to those reported before for CdSe/CdS and CdSe/ZnS core/shell NC systems. However, we do not observe the LO phonon peak related with the core, although it had been systematically observed earlier for CdSe-based core/shell NCs, as well as for CdTe NCs without CdS shell. The peculiarities of the electron-phonon coupling (EPC) in the strongly confined CdTe/CdS heterosystem may be the reason of such a different behavior and need further experimental and theoretical studies. In particular, much stronger EPC coupling (via Fröehlich mechanism) in CdS than in CdTe can be one of the reasons.

## Data Availability

The data presented in this study are available on request from the corresponding author.
